# A Novel Mouse *Fgfr2* Mutant, Hobbyhorse (*hob*), Exhibits Complete XY Gonadal Sex Reversal

**DOI:** 10.1371/journal.pone.0100447

**Published:** 2014-06-23

**Authors:** Pam Siggers, Gwenn-Aël Carré, Debora Bogani, Nick Warr, Sara Wells, Helen Hilton, Chris Esapa, Mohammad K. Hajihosseini, Andy Greenfield

**Affiliations:** 1 Mammalian Genetics Unit, Medical Research Council, Harwell, Oxfordshire, United Kingdom; 2 The Mary Lyon Centre, Medical Research Council, Harwell, Oxfordshire, United Kingdom; 3 School of Biological Sciences, University of East Anglia, Norwich, United Kingdom; John Hopkins University School of Medicine, United States of America

## Abstract

The secreted molecule fibroblast growth factor 9 (FGF9) plays a critical role in testis determination in the mouse. In embryonic gonadal somatic cells it is required for maintenance of SOX9 expression, a key determinant of Sertoli cell fate. Conditional gene targeting studies have identified FGFR2 as the main gonadal receptor for FGF9 during sex determination. However, such studies can be complicated by inefficient and variable deletion of floxed alleles, depending on the choice of Cre deleter strain. Here, we report a novel, constitutive allele of *Fgfr2*, hobbyhorse (*hob*), which was identified in an ENU-based forward genetic screen for novel testis-determining loci. *Fgr2^hob^* is caused by a C to T mutation in the invariant exon 7, resulting in a polypeptide with a mis-sense mutation at position 263 (Pro263Ser) in the third extracellular immunoglobulin-like domain of FGFR2. Mutant homozygous embryos show severe limb and lung defects and, when on the sensitised C57BL/6J (B6) genetic background, undergo complete XY gonadal sex reversal associated with failure to maintain expression of *Sox9*. Genetic crosses employing a null mutant of *Fgfr2* suggest that *Fgr2^hob^* is a hypomorphic allele, affecting both the FGFR2b and FGFR2c splice isoforms of the receptor. We exploited the consistent phenotype of this constitutive mutant by analysing MAPK signalling at the sex-determining stage of gonad development, but no significant abnormalities in mutant embryos were detected.

## Introduction

Fibroblast growth factors (FGF) function in numerous processes throughout embryonic development, such as the induction and patterning of germ cell layers, body axis formation and organogenesis [1]. Out of 22 human and mouse FGFs, 18 bind to a distinct set of cell-surface FGF receptors (FGFRs) to initiate intra-cellular signalling that results in cellular responses, including cell proliferation and differentiation [2]. Our understanding of the physiological roles of FGF ligands and their receptors has been helped enormously by the study of mouse knockouts [1]. Ranging from early embryonic lethality to adult metabolic abnormalities, these mutant phenotypes reveal the breadth of impact of FGF signalling.

Testis determination in the embryo normally requires the Y-linked gene *Sry* to initiate the commitment of somatic cells in the developing bipotential gonad to the Sertoli cell fate [3]. SRY effects this commitment through its positive effects on the expression of *Sox9*, a gene that is itself necessary and sufficient for testis development [4]. The analysis of mice lacking FGF9 first revealed a role for this signalling pathway in testis determination. *Fgf9*-deficient animals die around birth due to severe lung hypoplasia and, on a mixed genetic background, XY embryos exhibit a range of gonadal abnormalities ranging from testicular hypoplasia to complete sex reversal [5]. On the C57BL/6J (B6) background, which is sensitised to disruptions to testis determination, XY *Fgf9*-deficient embryos consistently exhibit gonadal sex reversal, indicating that some of the earliest processes in testis determination are disrupted by the absence of FGF9 [6]. Subsequent studies revealed an important role for FGF9 in maintaining high levels of *Sox9* expression in the developing XY gonad, mediated at least partly by its inhibitory effects on ovary-determining genes such as *Wnt4* [7], [8]. It has also been proposed that the rapid diffusion of secreted FGF9 along the long, thin gonad at around 11.5 dpc prevents any appreciable delay in the gonadal poles receiving the masculinising signal begun by expression of SRY at the centre of the gonad [9]. Any such delay may result in ovotestis or ovary development in an XY embryo due to the restricted time window that is thought to define the competence of cells to respond to SRY and its downstream effectors [10].

FGF9 acts as a paracrine FGF, mediating its effects locally by binding to and activating one of four tyrosine kinase FGFRs, using heparin sulphate proteoglycan (HSPG) cofactor-association as a means of regulating ligand distribution and receptor binding [11]. Loss-of-function genetic studies have identified FGFR2 as the likely receptor for embryonic gonadal FGF9. Embryos lacking FGFR2 die mid-gestation, at around 10.5 dpc, precluding a study of the effects of this loss on testis determination. Conditional gene targeting revealed partial XY gonadal sex reversal when *Fgfr2* deletion was restricted temporally, from around 10.5 dpc onwards, or spatially, to gonadal somatic cells [12]. Similar XY gonadal defects, including ovotestis and ovary development, were observed after mosaic deletion of *Fgfr2* in the embryo using *Ck19Cre* [13]. Limb and lung defects were also common in these embryos. Expression analysis in isolated pre-Sertoli cells indicates that one of the two isoforms of *Fgfr2*, *Fgfr2c*, is found in this lineage [12]. In contrast, both the *Fgfr2b* and *Fgfr2c* isoforms are detectable in other gonadal cells. Thus, these data suggest that FGFR2 is required for Sertoli cell differentiation and testis development through its activation by FGF9 ligand.

Despite the wealth of data concerning FGF signalling and its role in testis determination, the mechanism by which the gonadal FGF signal is transduced intracellularly remains unclear. In other contexts, a number of pathways are implicated, including PI3K-AKT and RAS-MAPK signalling [2]. Here, we report the identification of a novel, constitutive mutation of *Fgfr2*, hobbyhorse (*hob*). *Fgfr2^hob^* homozygotes exhibit severe lung hypoplasia and absence of limbs, in addition to complete XY gonadal sex reversal on the sensitised B6 background. We exploit the phenotypic robustness of this model of FGF-dependent sex reversal to address the role of MAPK signalling in transducing the FGF signal during testis determination.

## Materials and Methods

### Mouse strains used and genotyping

All animal experimentation was approved by the Animal Welfare and Ethical Review Body (AWERB) at MRC Harwell and mice used in this study were bred with licensed approval from the UK Home Office (PPL 30/2877). Mice were housed in individually ventilated cages (IVCs) in a specific pathogen-free (SPF) environment. Further details of micro- and macro-environmental conditions are available on request. Adult mice were humanely sacrificed by dislocation of the neck, confirmed by palpation, and embryos were decapitated in ice-cold, phosphate-buffered saline solution. Mice harbouring *Fgfr2^tm1.1Dor^* were generated by breeding a floxed allele of *Fgfr2* (*Fgfr2^tm1Dor^*/J, The Jackson Laboratory) with the ubiquitous Cre deleter *FVB/*N-T*g(AC*T*B-Cre)2Mrt/*J (The Jackson Laboratory) after at least three generations of backcrossing of each strain to C57BL/6J. Cre-mediated deletion of the floxed locus removes exons 7–10, generating a non-functional *Fgfr2* allele. Presence of the *Fgfr2^hob^* allele was detected with a LightScanner assay using the primers Fgfr2ex7F 5′-CCTTTCTCCATCAGAACGGTCA-3′ and Fgfr2ex7R 5′-CTGTAAACCTTGCAGACAAACTC-3′ with mutant probe: Fgfr2ex7 PrR 5′-GAGGCATTTGCAGACAGTCCAGCTT-3′.

Details of ENU mutagenesis, the three-generation breeding scheme and genome-wide mapping of developmental mutants identified have been described elsewhere [14]–[16]. Adult mice and embryos were sexed by a PCR assay that simultaneously amplifies the *Ube1y1* and *Ube1x* genes, using the following primer pair: 5′-TGGATGGTGTGGCCAATG-3′ and 5′-CACCTGCACGTTGCCCTT-3′ [17].

### Generation of embryos and expression analyses

Noon on the day of the copulatory plug was counted as 0.5 dpc. Embryos were staged accurately based on the number of tail somites (ts) or limb and gonad morphology. In the case of *hob/hob* embryos, somites were counted from the presumptive hindlimb bud, which appears as a thickening of the body wall in the area of the hindlimb field and is present up to approximately 12.5 dpc. Wholemount *in situ* hybridization (WMISH) analysis of embryonic tissues was performed as previously described [18]. Probes for *Sox9* [19], *Sry* [20], *Insl3* [17], *Wnt4* and *Stra8* [14], [21] have been previously described. WMISH was performed on at least three independent gonad samples from each embryonic class.

### Immunohistochemistry

Antibodies to the following proteins were utilised in this study: SOX9 (Millipore, #AB5535); AMH (Santa Cruz, #sc28912); FOXL2 (a kind gift from Dagmar Wilhelm and Peter Koopman [22]); SRY (a kind gift from Makoto Tachibana, Kyoto University [23]); Cell Signaling antibodies - p38 MAPK (CST9212), phospho-p38 MAPK (CST9215), ERK (CST9102) and phospho-ERK (CST4377); 12G10 alpha-tubulin (Developmental Studies Hybridoma Bank, University of Iowa). Immunostaining was performed on sectioned, paraffin wax-embedded tissue from two independent gonad samples using the above primary antibodies (1∶100) and Alexa Fluor 594 (AMH, SOX9) or 488 (FOXL2, SRY) conjugated secondary antibodies (1∶200). Images were captured using a Zeiss 710 multiphoton microscope.

### Protein detection by Simple Western analysis

Pooled sub-dissected gonads (n = 8) were lysed using 50 µl RIPA buffer (150 mM NaCl, 1% NP40, 0.5% sodium deoxycholate, 0.1% SDS, 50 mM Tris, pH 7.5) supplemented with Aqueous and DMSO inhibitor cocktails (Protein Simple), and cell debris removed by centrifugation. Lysates (7.5 µl) were mixed with 2.5 µl of Simple Western sample dilution buffer (Protein Simple) containing reducing agent and fluorescent standards, and denatured at 95°C for 5 min. Lysates (10 µl), primary antibodies, horseradish peroxidase–conjugated secondary antibodies, separation matrix, stacking matrix and chemiluminescent substrate were dispensed into designated wells in a 384-well assay plate. Simple Western assay buffers, capillaries and the prepared assay plate were placed in Peggy instrument (Protein Simple), which carries out all assay steps automatically for up to 8 cycles. Briefly, proteins were separated through a size-resolving matrix in capillaries, immobilized to the inner capillary wall, incubated with primary and secondary antibodies before detection using chemiluminescence. Signal and quantitation of immunodetected proteins were generated automatically at the end of the run. Statistical analysis was performed on data from three biological replicates (24 gonad samples in total).

## Results

### Identification of the hobbyhorse (*hob*) mutation: an allele of *Fgfr2* that disrupts limb, lung and gonad development

**Figure 1:**
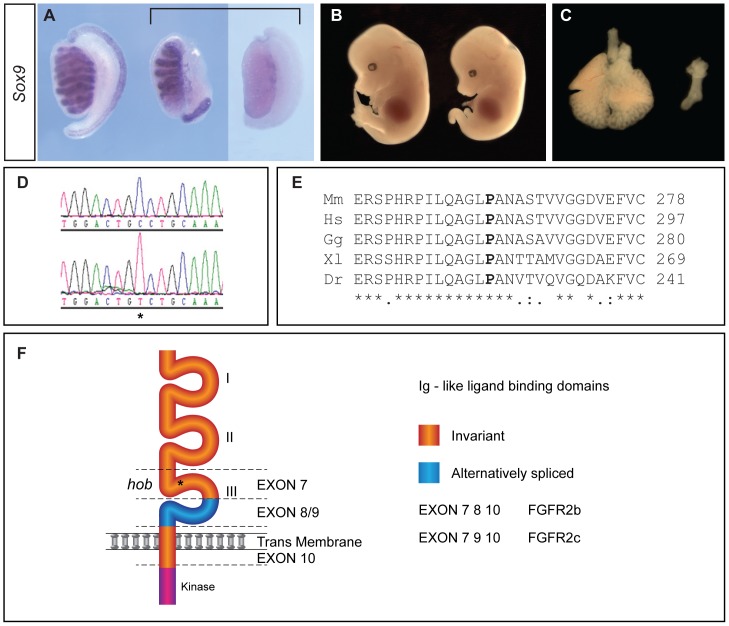
The hobbyhorse (*hob*) mutation disrupts XY sex determination and is caused by an ENU-induced point mutation of *Fgfr2*. A) A wild-type XY gonad (left) showing characteristic testicular morphology at 14.5 dpc, in contrast to two XY hobbyhorse mutants identified in a forward genetic screen, which have disrupted testis cords (centre) or lack cords entirely (right). All gonads shown are after wholemount *in situ* hybridisation (WMISH) with a *Sox9* probe. B) A hobbyhorse mutant (right) lacks limbs. A wild-type embryo is also shown (left). C) Absence of lung development in a hobbyhorse embryo (right), in contrast to normal lungs at the same stage (left). D) Sequence trace showing homozygosity for a C to T mutation (asterisk) in exon 7 of *Fgfr2* of a hobbyhorse embryo. Upper trace is wild-type, lower trace is hobbyhorse. E) The proline residue that is mutated in the *hob* allele is highly conserved in vertebrates. Mm, *Mus musculus*; Hs, *Homo sapiens*; Gg, *Gallus gallus*; Xl, *Xenopus leavis*; Dr, *Danio rerio*. F) Diagrammatic representation of FGFR2 and its domain structure in the FGFR2b and FGFR2c isoforms. The *hob* mutation (asterisk) resides in the third extracellular immunoglobulin-like domain, encoded by the invariant exon 7.

We have previously described a forward genetic screen employing *N*-ethyl-*N*-nitrosourea (ENU) mutagenesis aimed at identifying novel loci required for mouse development. This screen identified recessive mutations in genes functioning in neural tube closure and patterning [15], left-right patterning [16] and sex determination [14]. We identified a second pedigree in this screen (RECB/135) containing embryos with gonadal abnormalities at 14.5 dpc. Affected XY gonads exhibited a range of morphological abnormalities, ranging from disruption to cord formation through to complete absence of testis cords (Fig 1A). This variability is likely to be due to the mixed genetic background on which the screen was performed. Affected embryos also lacked limbs and had severely hypoplastic lungs (Fig 1B,C); due to their external appearance the mutants were called hobbyhorse (*hob*).

Using seven affected embryos and a genome-wide panel of polymorphic markers, we mapped the mutation responsible for this phenotype to distal mouse chromosome 7, between the markers rs6317573 and rs32097269 (data not shown). The phenotypic combination of lung, limb and gonadal abnormalities was strongly reminiscent of defects in FGF/FGFR signalling previously reported [5], [12], [13], and inspection of the critical linkage region identified the *Fgfr2* gene. We amplified and sequenced *Fgfr2* exons in genomic DNA from affected and unaffected embryos and identified a homozygous C to T mutation in exon 7 of all, and only, affected individuals (Fig 1D). This mutation results in a predicted proline to serine mis-sense amino acid substitution at position 263 (P263S) of the FGFR2 polypeptide chain. The affected proline residue is highly conserved in vertebrates (Fig1E) and resides in the third extracellular immunoglobulin-like domain (IgIII, D3) of FGFR2 (Fig 1F). Moreover, exon 7 is an invariant exon in *Fgfr2* mRNA isoforms and so this mutation is predicted to affect both the *Fgfr2b* and *Fgfr2c* isoforms. The extracellular region of FGFRs encompassing the second and third Ig-like domains (D2, D3) and the intervening linker region is necessary and sufficient for FGF ligand binding [24]. Specificity of ligand binding by FGFR2 is determined by alternate splicing in the D3 domain.

### Complete gonadal sex reversal in XY *Fgfr2^hob/hob^* embryos on C57BL/6J

**Figure 2:**
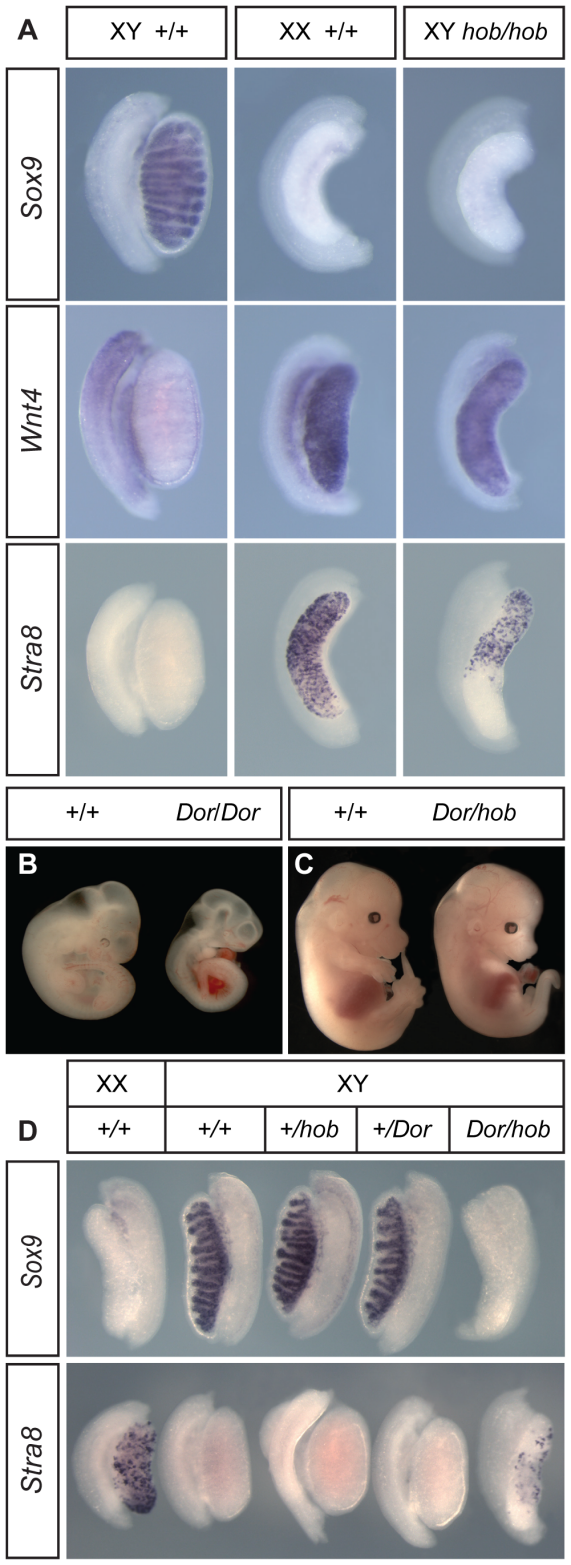
Characterisation of XY *Fgfr2^hob/hob^* embryonic gonad development on the C57BL/6J (B6) background and complementation test with the *Fgfr2^tm1.1Dor^* null allele. A) WMISH analysis of gonads at 14.5 dpc from XY wild-type, XX wild-type and XY *Fgfr2^hob/hob^* embryos using a marker of the Sertoli cell lineage (*Sox9*), ovarian somatic cells (*Wnt4*) and meiotic germ cells (*Stra8*). B) Embryos homozygous for the *Fgfr2^tm1.1Dor^* allele (*Dor/Dor*) are much smaller than wild-type controls (+/+) at 11.5 dpc and also lack limbs. C) Embryos at 14.5 dpc doubly heterozygous for the *Fgfr2^hob^* and *Fgfr2^tm1.1Dor^* alleles (*Dor/hob*) lack limbs and are noticeably smaller than wild-type controls (+/+). D) Upper panel: *Sox9* WMISH of 13.5 dpc embryonic gonads from control and XY *Fgfr2^tm1.1Dor/hob^* doubly heterozygous embryos; lower panel: *Stra8* WMISH of 14.5 gonads from embryos of same genotypes as upper panel. The developmental stage of the doubly heterozygous gonad in the lower panel appears significantly retarded when compared to the XX control.

We crossed the *Fgfr2^hob^* mutation to C57BL/6J (B6) for at least three generations to remove contaminating mutations and to examine gonad development in homozygotes on this sensitised genetic background. Adult heterozygotes were fertile and exhibited no overt abnormalities, suggesting that *Fgfr2^hob^* is a genuine recessive mutation. B6 XY embryos homozygous for the *Fgfr2^hob^* mutation exhibited complete gonadal sex reversal, as evidenced by the ovarian morphology and the absence of the Sertoli cell marker, *Sox9*, at 14.5 dpc and the high levels of the ovarian somatic marker *Wnt4* (Fig 2A); in addition, expression of *Stra8* was also observed in XY mutant gonads, indicating entry of germ cells into meiosis and thus germ cell sex-reversal (Fig 2A). Homozygous embryos still lacked limbs; a presumptive hindlimb bud, which appears as a thickening of the body wall in the area of the hindlimb field, was present up to approximately 12.5 dpc, as previously described in some embryos homozygous for a null allele of *Fgfr2* (data not shown) [25]. Homozygous embryos also had hypoplastic lungs (data not shown) but were otherwise viable until late in gestation. In order to confirm that homozygosity for *Fgfr2^hob^* alone accounted for the mutant phenotype, we performed a genetic complementation test with an *Fgfr2* null allele (*Fgfr2^tm1.1Dor^*). We first confirmed that embryos homozygous for *Fgfr2^tm1.1Dor^* were dying from around 10.5–11.0 dpc and lacked limb buds (Fig 2B). We then generated embryos carrying a single copy of both *Fgfr2^hob^* and *Fgfr2^tm1.1Dor^*. In contrast to *Fgfr2^tm1.1Dor^* homozygotes, doubly heterozygous embryos were alive at 14.5 dpc, though they were slightly smaller than wild-type littermates and had no limbs and exhibited lung hypoplasia (Fig 2C and data not shown). XY gonads from doubly heterozygous embryos had an ovarian morphology and exhibited negligible *Sox9* expression at 13.5 dpc and *Stra8*-positive germ cells at 14.5 dpc (Fig 2D). From this complementation assay we conclude that homozygosity for the *Fgfr2^hob^* mutation accounts for limb, lung and gonadal abnormalities identified in the RECB/135 pedigree. Moreover, based on the fact that *Fgfr2^hob/m1.1Dor^* double heterozygotes survive for longer than *Fgfr2^tm1.1Dor^* homozygotes, we infer that *Fgfr2^hob^* is a hypomorphic allele. Genetic interaction tests involving *Fgfr2^hob^* and a null allele of *Map3k4* [14] did not, in contrast, generate embryonic gonads with overt abnormalities (Fig S1).

### Cellular and molecular phenotyping of XY *Fgfr2^hob/hob^* gonads

**Figure 3:**
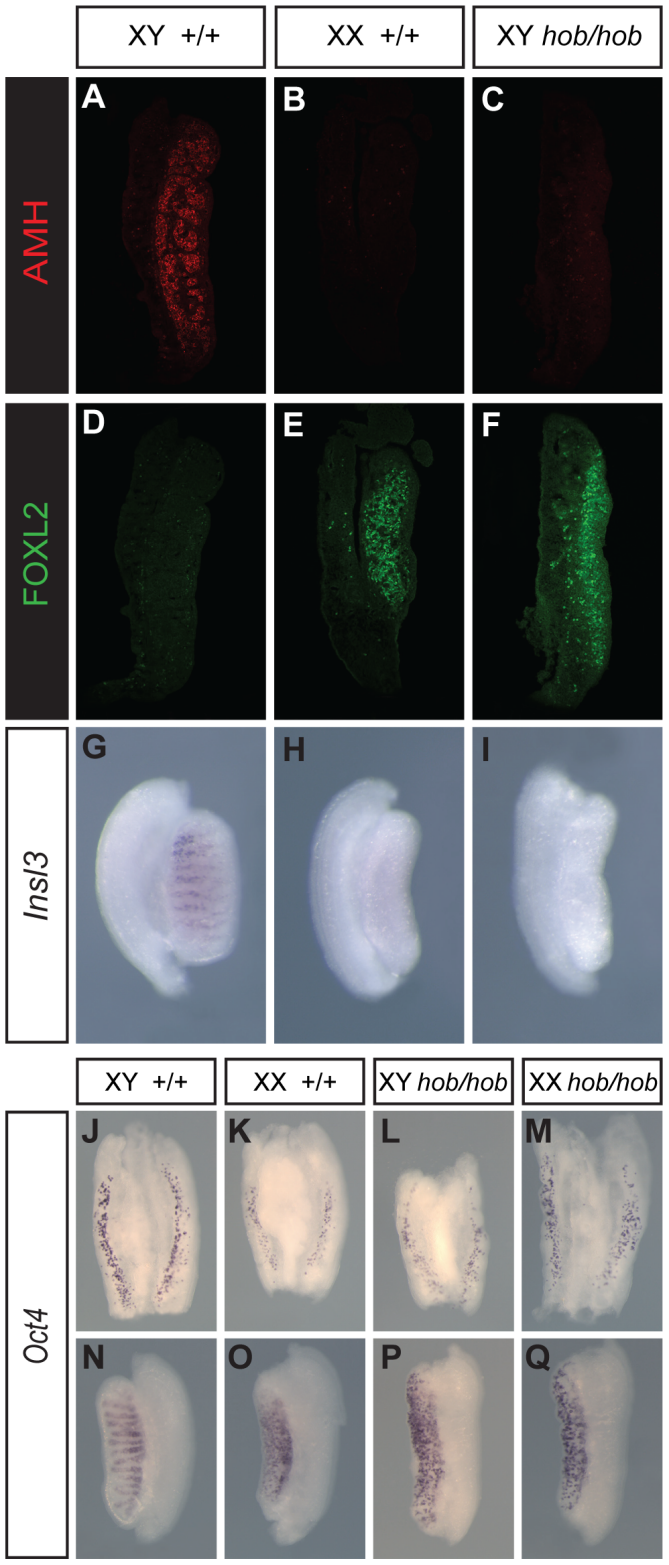
Complete XY gonadal sex reversal in *Fgfr2^hob/hob^* embryos on B6. A–C) Immunostaining with anti-AMH antibody of gonadal sections from XY wild-type (A), XX wild-type (B) and XY *Fgfr2^hob/hob^* (C) embryos at 12.5 dpc. D–F) anti-FOXL2 immunostaining of samples equivalent to those in A–C. G–I) WMISH for *Insl3* (a marker of Leydig cells) of gonads with same genotypes as A–C. J–M) *Oct4* WMISH of 11.5 dpc (17 ts) gonads from control XY (J), XX (K), XY *Fgfr2^hob/hob^* and XX *Fgfr2^hob/hob^* gonads. N–Q) *Oct4* WMISH of 13.5 dpc gonads from embryos of the same genotype as J-M.

We then performed a more detailed analysis of gonad development in XY *Fgfr2^hob/hob^* homozygous embryos on B6 between 11.5 dpc and 14.5 dpc. Immunostaining revealed anti-Müllerian hormone (AMH), a marker of Sertoli cells, in the testis cords of wild-type gonads at 12.5 dpc, but none was detected in mutant gonadal tissue (Fig 3A–C). In contrast, the ovarian somatic marker FOXL2 was detected in mutant gonads at 12.5 dpc, but not in wild-type XY controls (Fig 3D–F). Sex reversal extended to the steroidogenic lineage, with no *Insl3* transcript detected in XY mutants at 14.5 dpc (Fig 3G–I). However, *Oct4* expression was detectable in both XY and XX *Fgfr2^hob/hob^* homozygotes at 11.5 dpc (Fig 3J–M) and 13.5 dpc (Fig 3N–Q), suggesting no overt disruption to germ cell development at this stage.

**Figure 4:**
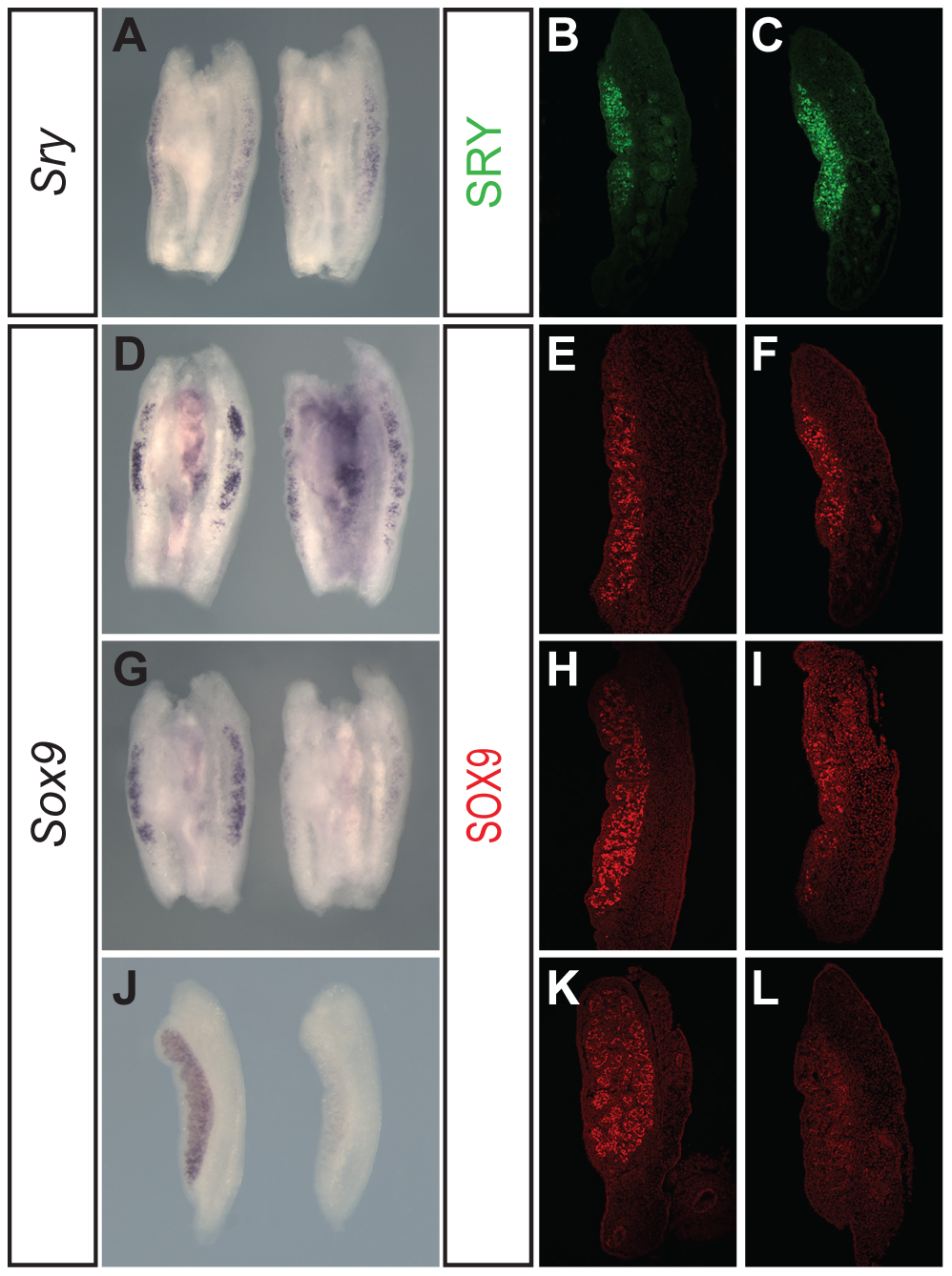
Normal *Sry* expression, but disrupted *Sox9* expression, in XY *Fgfr2^hob/hob^* embryonic gonads. A) *Sry* WMISH at 11.5 dpc (16 ts) showing expression in XY wild-type (left) and XY *Fgfr2^hob/hob^* (right) gonads. B, C) anti-SRY immunostaining at 18 ts in wild-type (B) and XY *Fgfr2^hob/hob^* (C) gonads. D) *Sox9* WMISH at 18 ts with tissue samples as described in (A). E, F) anti-SOX9 immunostaining at 18 ts in XY wild-type (E) and XY *Fgfr2^hob/hob^* (F) gonads. G) *Sox9* WMISH at 23 ts in XY wild-type (left) and XY *Fgfr2^hob/hob^* (right) gonads. H, I) anti-SOX9 immunostaining at 23 ts in XY wild-type (H) and XY *Fgfr2^hob/hob^* (I) gonads. J) *Sox9* WMISH at 12.5 dpc in XY wild-type (left) and XY *Fgfr2^hob/hob^* (right) gonads. K, L) anti-SOX9 immunostaining at 13.0 dpc in wild-type (K) and XY *Fgfr2^hob/hob^* (L) gonads.

Previously, loss of FGF9 has been associated with disruption to *Sox9* expression, but *Sry* expression has been reported to be unaffected [7]. We first examined *Sry* in XY *Fgfr2^hob/hob^* embryos using WMISH and anti-SRY immunostaining of gonads at 11.5 dpc (from 16–18 tail somites (ts)). Neither of these methods revealed any disruption to *Sry* expression (Fig 4A–C). This is in agreement with a previous report on one *Fgfr2* CKO [13]. In contrast, *Sox9* expression, as assayed by WMISH and immunostaining, whilst initially up-regulated at 18 ts (Fig 4D–F), was observed at greatly reduced levels at 23 ts (around 11.75 dpc) in mutant gonads (Fig 4G–I) and at 12.5–13.0 dpc (Fig 4J–L), consistent with data reported concerning *Sox9* expression in *Fgf9*-deficient gonads [7]. Thus, data concerning the molecular basis of sex reversal in XY *Fgfr2^hob^* homozygotes indicate normal up-regulation of *Sox9*, but a failure to maintain significant levels beyond 11.5 dpc.

### MAPK signalling in *Fgfr2^hob/hob^* homozygous gonads at 11.5 dpc

**Figure 5:**
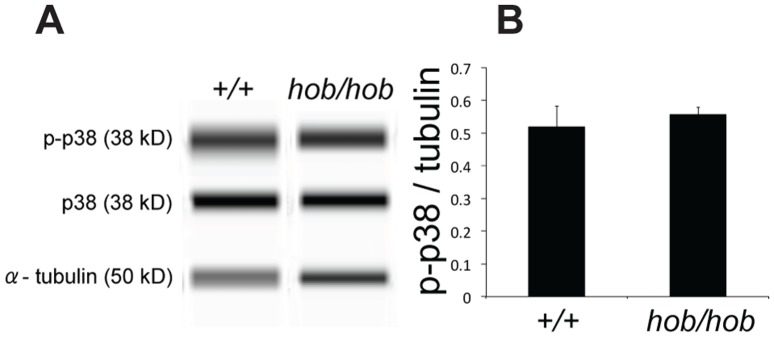
Quantitation of phospho-p38 MAPK (p-p38) levels in gonadal samples at 11.5 dpc (16–18 ts) in XY wild-type and *Fgfr2^hob/hob^* gonads. A) Lane view images showing Simple Western detection of p-p38, p38, and α-tubulin. B) Graph showing the ratio of p-p38 to tubulin in the two gonadal genotypes. The ratio of p-p38 to p38 was similarly unaltered. Errors were calculated using standard error mean.

The FGF signal is transduced intra-cellularly by means of a number of pathways, including PI3K-AKT and RAS-MAPK signalling [2]. Based on the profile of *Sox9* expression in mutant gonads, which indicates defective expression from around 11.75 dpc, we performed a quantitative analysis of MAPK signalling in *Fgfr2^hob/hob^* homozygous gonads at around 11.5 dpc (16–18 ts) in order to identify potential abnormalities in signalling that might result in subsequent defects in *Sox9* expression. Gonadal tissue from XY and XY*^hob/hob^* embryos was dissected away from the mesonephros and protein lysates were examined using a size-based, capillary electrophoresis method for immunodetection of the phosphorylated and non-phosphorylated forms of p38 MAPK and ERK 1/2. Both of these MAPKs are activated in developmental contexts by the FGF signal [26], [27]. At 16–18 ts no significant differences were observed between control and mutant gonadal tissue samples in the levels of phospho-p38 MAPK (Fig 5A, B). Unfortunately, we were unable to detect p-ERK at this stage in gonadal tissue, despite the fact that ERK itself was detectable and control samples from cultured cells indicated that the anti-p-ERK antibody was working reliably (data not shown).

## Discussion

Here we report the identification, in a mouse forward genetic screen, of a novel sex-reversing mutant allele of *Fgfr2*. Previous studies of *Fgfr2* function in testis determination have relied on conditional gene targeting, with the attendant variability in efficiency and site of gene ablation resulting in some phenotypic variation. Homozygous deletion of floxed *Fgfr2* alleles using *Sf1*-Cre, which is expressed primarily in somatic cells within the gonad, resulted in partial XY sex reversal, with ovotestis formation commonly observed at 15.5 dpc [12]. Deletion of *Fgfr2* with *Ck19*:Cre, a line that deletes in epiblast-derived cells in a mosaic fashion, also resulted in variable and partial XY gonadal sex reversal [13]. Complete sex reversal was observed after *Fgfr2* deletion at 10.5 dpc with a heat-shock inducible Cre line, *Hs-Cre*, but there were variable numbers of SOX9-positive cells observed in mutant gonads [12]. Incomplete deletion of floxed alleles may contribute to phenotypic variability, as might residual genetic background variation. The constitutive *Fgfr2^hob^* allele affords a detailed study of the role of FGFR2 in mouse sex determination on a stable genetic and phenotypic background. The value of forward genetics in the identification of developmental loci, including the provision of new and useful alleles of genes of known function, is underlined by this study.

Our data suggest that *Fgfr2^hob^* is a hypomorphic mutant allele, perhaps disrupting interaction of FGFR2 with its ligand, given the position of the altered amino acid in the extracellular immunoglobulin-like domain. This prediction requires testing in a future study. The extended viability of *Fgfr2^hob/hob^* embryos, in comparison to *Fgfr2^tm1.1Dor/tm1.1Dor^* homozygotes that completely lack FGFR2 function, is also suggestive of an overcoming of an early placental defect in *Fgfr2^hob/hob^* embryos. It is this placental defect that is thought to account for the death of *Fgfr2^tm1.1Dor/tm1.1Dor^* homozygotes at around 11.5 dpc. The amelioration of this placental defect is in contrast to the persistent absence of limbs and severe lung hypoplasia that is a common phenotypic feature of homozygotes for both alleles. This discrepancy might be explained by a lower threshold of FGFR2 function in the developing placenta, greater functional redundancy with respect to FGF receptor signalling in this tissue or distinct ligand sensitivities to the *hob* mutation. With respect to the relative importance of the *Fgfr2b* and *Fgfr2c* isoforms in testis determination, our data do not allow us to discriminate because we predict that the *hob* allele disrupts both isoforms of the receptor. It will be important to carefully analyse gonad development in mice specifically lacking either the Fgfr2b or Fgfr2c isoform on the B6 genetic background in order to determine their individual contributions to testis determination.

Gonadal sex reversal in XY *Fgfr2^hob/hob^* embryos is associated with a failure to maintain high levels of the key testis determinant, SOX9, after 11.5 dpc. In contrast, the pro-ovarian gene *Wnt4* is elevated, indicating sex reversal of the supporting cell lineage. These data are consistent with observations of failure to maintain *Sox9* expression in *Fgf9*-deficient XY gonads [7]. In contrast, *Sry* expression appears to remain unaffected by disruption of FGF9/FGFR2 signalling. Recent studies involving phenotypic analysis of XY embryos lacking *Fgf9* and *Wnt4* suggest that the primary role of FGF9 may be to inhibit the activity of WNT/β-catenin signalling [8]. The mechanistic basis of this at the molecular level remains unclear, but *Fgfr2^hob/hob^* embryos may offer a useful tool for investigating such antagonistic interactions of the testis- and ovary-determining gene regulatory networks (Carre & Greenfield). However, an inhibitory role for FGF9/FGFR2 signalling may suggest that the search for targets of FGF-dependent signalling in the gonad should be extended to proteins associated with ovary development and canonical WNT signalling more generally.

We have exploited the relative stability of the XY *Fgfr2^hob/hob^* embryonic phenotype to perform careful quantitation of MAPK signalling during the testis-determining stages of gonadogenesis and report no significant deficits at early stages (16–18 ts). We have previously reported a role for p38 MAPK signalling in regulation of *Sry* expression and testis determination [28]. The unaltered levels of phospho-p38 MAPK at 16-18 ts in *Fgfr2^hob/hob^* embryonic gonads are therefore consistent with the observation of normal *Sry* expression in this sex-reversing mutant. Technical improvements may be required to detect any p-ERK present and thereby determine whether a role exists for this MAPK in regulating expression of *Sox9* during testis determination, as reported in other contexts [29]. It will also be important to analyse the phosphorylation status of any transcription factor implicated in regulation of *Sox9* or *Wnt4* expression in *Fgfr2^hob/hob^* embryos, or cellular models derived from these, in future studies.

## Supporting Information

Figure S1 **Absence of genetic interaction between** ***Fgfr2^hob^ (hob)*** **and** ***Map3k4^tm1Flv^*** **(** ***M4*** **).** *Stra8* WMISH of control gonads and gonads from doubly heterozygous embryos (+*/hob*, +*/M4*). Expression is only detected in control XX gonads.
(TIF)
