# Supplementary figures and images for: A Novel Mouse Fgfr2 Mutant, Hobbyhorse (hob), Exhibits Complete XY Gonadal Sex Reversal
Source: PLoS One. 2014 Jun 23;9(6):e100447. doi: 10.1371/journal.pone.0100447 (PMC4067367; doi:10.1371/journal.pone.0100447)

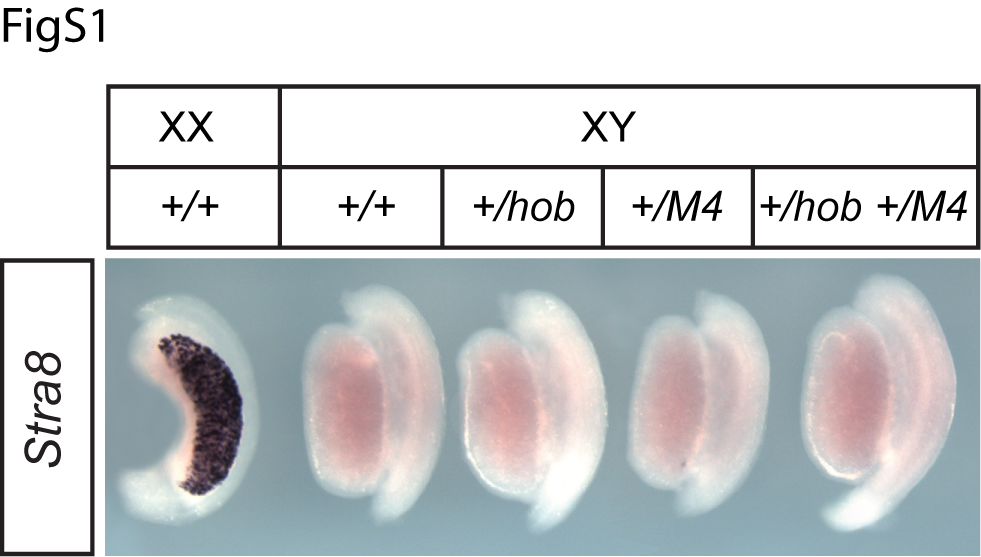

Supplement: Figure S1 — Absence of genetic interaction between Fgfr2hob (hob) and Map3k4tm1Flv ( M4 ). Stra8 WMISH of control gonads and gonads from doubly heterozygous embryos (+/hob, +/M4). Expression is only detected in control XX gonads. (TIF) [file pone.0100447.s001.tif]
